# Prognostic significance of preoperative prognostic nutritional index in colorectal cancer: results from a retrospective cohort study and a meta-analysis

**DOI:** 10.18632/oncotarget.10148

**Published:** 2016-06-17

**Authors:** Yuchong Yang, Peng Gao, Xiaowan Chen, Yongxi Song, Jinxin Shi, Junhua Zhao, Jingxu Sun, Yingying Xu, Zhenning Wang

**Affiliations:** ^1^ Department of Surgical Oncology and General Surgery, First Hospital of China Medical University, Shenyang City, PR China; ^2^ Department of Breast Surgery, First Hospital of China Medical University, Shenyang City, PR China

**Keywords:** colorectal cancer, meta-analysis, prognostic nutritional index, prognosis, TNM staging

## Abstract

The preoperative prognostic nutritional index (PNI) may forecast colorectal cancer (CRC) outcomes, but the evidence is not conclusive. Here, we retrospectively analyzed a cohort of patients from the Department of Surgical Oncology at the First Hospital of China Medical University (CMU-SO). We also conducted a meta-analysis of eleven cohort studies. Bayesian Information Criterion (BIC) was used to determine the optimal PNI cut-off values for classifying prognosis in the patients from the CMU-SO. The result from CMU-SO and meta-analysis both confirmed that low PNI was significantly associated with a poor prognosis and advanced TNM stages. Among the patients from the CMU-SO, the optimal cut-off values were “41-45-58” (PNI < 41, 41 ≤ PNI < 45, 45 ≤ PNI < 58, PNI ≥ 58), which divided patients into 4 stages. The BIC value for TNM staging combined with the PNI was smaller than that of TNM staging alone (−325.76 *vs*. −310.80). In conclusion, low PNI was predictive of a poor prognosis and was associated with clinicopathological features in patients with CRC, and the 41-45-58 four-stage division may be suitable for determining prognosis. PNI may thus provide an additional index for use along with the current TNM staging system to determine more accurate CRC prognoses.

## INTRODUCTION

Colorectal cancer (CRC) is the second most common cancer in women and third most common in men; an estimated 1.4 million cases and 693,900 deaths occurred in 2012 [1]. Despite advancements in surgery, adjuvant chemoradiotherapy and targeted therapy [2, 3], the prognosis of CRC patients remains unsatisfactory. At present, TNM staging is considered the primary prognostic indicator. However, TNM staging is limited because patients with the same stage may have different clinical outcomes. Therefore, a new more accurate prognostic indicator for CRC patients is required to improve prognostic accuracy.

Some studies have reported that the progression and prognosis of cancer are determined not only by tumor features but also by nutritional and immunological conditions [4–7]. The prognostic nutritional index (PNI), calculated from serum albumin levels and peripheral lymphocyte count, reflects both the nutritional and immune status of the patient [8, 9]. Many recent studies demonstrate that PNI is a significant prognostic indicator for some malignancies [10], including hepatocellular carcinoma [11], pancreatic cancer [12], laryngeal cancer [13], renal cell carcinoma [14], and gastric carcinoma [15]. Although several studies on PNI have evaluated CRC prognosis [16, 17], few pooled studies and few studies with large sample size have explored the prognostic role of PNI in patients with CRC. Moreover, to the best of our knowledge, no studies have focused on the use of PNI as an additional index on the basis of the current TNM staging system. On the other hand, controversy still exists concerning the optimal cut-off values and how groups should be classified by PNI staging to determine CRC prognosis.

In this study, we retrospectively analyzed a cohort of patients from the Department of Surgical Oncology at the First Hospital of China Medical University (CMU-SO). We also conducted a meta-analysis with eleven cohort studies. In addition, we aimed to identify the optimal cut-off values and the most suitable divisions by PNI staging for determining prognosis in patients with CRC.

## RESULTS

Most relevant studies divide PNI into two groups to study its prognostic value. We split PNI into two groups according to the BIC method, allowing the comparison of the results from the meta-analysis. In patients from the CMU-SO, the median PNI was 51.3 (range, 32.3-71.2). We calculated Bayesian information criterion (BIC) values using different cut-off values; when the cut-off value was set at 45, the BIC values were the smallest for overall survival (OS) and cause-specific survival (CSS) (−9.527 and −8.427, respectively), indicating that the optimal cut-off value for splitting into two groups was 45. Of the 2062 patients evaluated, 275 patients (13.34%) with a PNI < 45 and 1787 patients (86.66%) with a PNI ≥ 45 were classified into the low PNI and high PNI groups, respectively.

### PNI and clinicopathological features

For patients from the CMU-SO, low PNI was significantly associated with older age, larger tumor size, tumor location in the colon, poor differentiation, increased tumor depth, advanced TNM stages, and fewer patients on postoperative chemotherapy. There was no significant difference in the sexes (*P* = 0.193), lymph node metastasis status (*P* = 0.276), or distant metastasis status (*P* = 0.072) between the low- and high-PNI groups (Table 1).

**Table 1:** Associations of PNI status with clinicopathological features in CRC patients from CMU-SO

		PNI status				
Variable	Number (%)	High PNI (%)	Low PNI (%)		*P*	
Sample size	2062 (100)	1787 (86.7)	275 (13.3)			
Age, Mean±SD, y		60.7±10.5	65.0±10.0		<0.001	
Gender					0.193	
Male	1166 (56.5)	997 (55.8)	169 (61.5)			
Female	896 (43.5)	790 (44.2)	106 (38.5)			
Tumor size (cm)					<0.001	
≥4.6	1035 (50.2)	952 (53.3)	200 (72.7)			
<4.6	1027 (49.8)	835 (46.7)	75 (27.3)			
Tumor location					<0.001	
Colon	871 (42.2)	701 (39.2)	170 (61.8)			
Rectum	1191 (57.8)	1086 (60.8)	105 (38.2)			
Differentiation					0.002	
Well - moderate	1885 (91.4)	1648 (92.2)	237 (86.2)			
Poor - undifferentiated	177 (8.6)	139 (7.8)	38 (13.8)			
pT category					0.001	
T1	58 (2.8)	54 (3.0)	4 (1.6)			
T2	381 (18.5)	352 (19.7)	29 (10.5)			
T3	852 (41.3)	722 (40.4)	130 (47.3)			
T4	771 (37.4)	659 (36.9)	112 (40.7)			
pN category					0.276	
pN0	1216 (59.0)	1058 (59.2)	158 (57.5)			
pN1	619 (30.0)	540 (30.2)	79 (28.7)			
pN2	227 (11.0)	189 (10.6)	38 (13.8)			
Distant metastasis					0.072	
Negative	2018 (97.9)	1753 (98.1)	265 (96.4)			
Positive	44 (2.1)	34 (1.9)	10 (3.6)			
TNM stage					0.001	
I	351 (17.0)	326 (18.2)	25 (9.1)			
II	855 (41.5)	726 (40.6)	129 (46.9)			
III	812 (39.4)	701 (39.2)	111 (40.4)			
IV	44 (2.1)	34 (1.9)	10 (3.6)			
Postoperative chemotherapy					<0.001	
Absent	886 (43.0)	732 (41.0)	154 (56.0)			
Present	1176 (57.0)	1055 (59.0)	121 (44.0)			

Abbreviations, PNI: prognostic nutritional index; SD: standard deviation.

For patients from the meta-analysis, there were significantly more patients with older age, positive lymph node metastasis, advanced TNM stages, postoperative complications, and who did not receive postoperative chemotherapy in the low-PNI group than in the high-PNI group. The differences in tumor depth (*P* = 0.172) and tumor differentiation (*P* = 0.131) were not significant between the two groups (Table S1).

### PNI and prognosis

**Figure 1:**
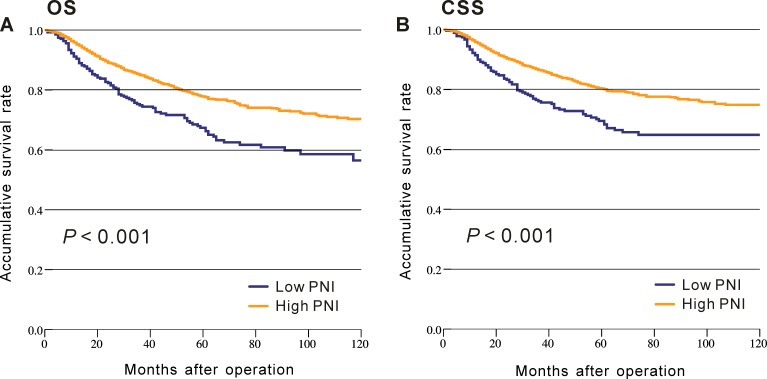
Kaplan-Meier analysis of survival based on low and high prognostic nutritional index among patients from CMU-SO **A.** overall survival; **B.** cancer-specific survival.

**Figure 2:**
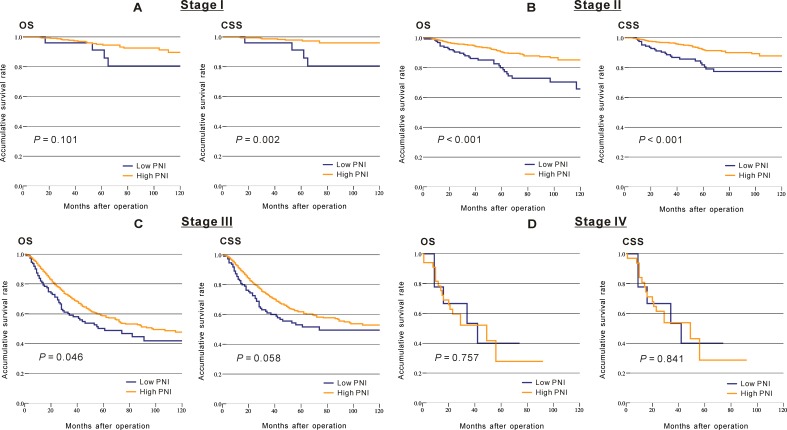
Kaplan-Meier analysis of the overall survival (OS) and cancer-specific survival (CSS) based on low and high prognostic nutritional index among patients from CMU-SO with stage I (**A.**, OS, *P* = 0.101; CSS, *P* = 0.002), stage II (**B.**, OS, *P* < 0.001; CSS, *P* < 0.001), stage III (**C.**, OS, *P* = 0.046; CSS, *P* = 0.058) and stage IV (**D.**, OS, *P* = 0.757; CSS, *P* = 0.841).

In the patients from the CMU-SO, the 5-year OS rate was 67.4 % in the low-PNI group and 77.8% in the high-PNI group (*P* < 0.001, Figure 1A, Table 2). The 5-year CSS rate was 69.5 % in the low-PNI group and 80.3% in the high-PNI group (*P* < 0.001, Figure 1B, Table 2). Furthermore, subgroup analysis shows the relationship of PNI with patient prognosis at each stage. OS was significantly different between the two groups at stages II and III (*P* < 0.001, *P* = 0.046) but not at stages I and IV (*P* = 0.101, *P* = 0.757, Figure 2). CSS was significantly different between the groups at stages I and II (*P* = 0.002, *P* < 0.001) but not at stages III and IV (*P* = 0.058, *P* = 0.841, Figure 2). In addition, Cox multivariate analysis indicates that low PNI was a poor independent prognostic factor for OS (HR = 1.282, 95% CI = 1.020-1.610, *P* = 0.033) and CSS (HR = 1.343, 95% CI = 1.051-1.715, *P* = 0.018, Table 2).

**Table 2:** Univariate and multivariate survival analyses of OS and CSS in CRC patients from CMU-SO

	Overall survival				Cancer-Specific Survival			
	Univariate		Multivariate		Univariate		Multivariate	
Variable	HR (95% CI)	*P*	HR (95% CI)	*P*	HR (95% CI)	*P*	HR (95% CI)	*P*
Gender								
Female	1				1			
Male	1.224 (1.020-1.469)	0.030			1.158 (0.953-1.408)	0.141		
Age (y)								
≥62	1		1		1			
<62	0.761 (0.636-0.911)	0.003	0.778 (0.645-0.938)	0.009	0.877 (0.723-1.062)	0.179		
Tumor Size (cm)								
≥4.6	1				1			
<4.6	0.894 (0.748-1.068)	0.217			0.888 (0.733-1.076)	0.224		
Tumor location								
Colon	1				1			
Rectum	1.078 (0.899-1.292)	0.418			1.104 (0.908-1.343)	0.321		
Differentiation								
Well - moderate	1		1		1		1	
Poor - undifferentiated	2.252 (1.745-2.906)	<0.001	1.574 (1.213-2.041)	0.001	2.317 (1.766-3.041)	<0.001	1.532 (1.162-2.021)	0.003
pT category								
T1	1		1		1		1	
T2	2.548 (0.795-8.168)	0.116	2.404 (0.749-7.715)	0.140	1.833 (0.565-5.952)	0.313	1.699 (0.522-5.523)	0.378
T3	5.321 (1.703-16.623)	0.004	3.098 (0.987-9.719)	0.053	4.500 (1.439-14.076)	0.010	2.445 (0.777-7.690)	0.126
T4	7.645 (2.444-23.919)	<0.001	4.507 (1.432-14.189)	0.010	6.779 (2.165-21.224)	0.001	3.659 (1.160-11.540)	0.027
pN category								
pN0	1		1		1		1	
pN1	4.355 (3.492-5.430)	<0.001	4.299 (3.426-5.394)	<0.001	5.020 (3.916-6.434)	<0.001	4.812 (3.731-6.207)	<0.001
pN2	10.349 (8.085-13.246)	<0.001	9.954 (7.687-12.890)	<0.001	12.722 (9.707-16.675)	<0.001	11.612 (8.759-15.394)	<0.001
Distant metastasis								
Negative	1		1		1		1	
Positive	3.818 (2.484-5.867)	<0.001	2.450 (1.581-3.797)	<0.001	4.143 (2.666-6.437)	<0.001	2.362 (1.511-3.695)	<0.001
TNM stage								
I	1				1			
II	1.876 (1.190-2.959)	0.007			2.598 (1.442-4.682)	0.001		
III	8.791 (5.761-13.414)	<0.001			13.727 (7.878-23.918)	<0.001		
IV	16.109 (8.956-28.975)	<0.001			26.243 (13.112-52.525)	<0.001		
Postoperative chemotherapy								
Absent	1		1		1		1	
Present	0.697 (0.583-0.834)	<0.001	0.497 (0.410-0.602)	<0.001	0.795 (0.656-0.963)	0.019	0.522 (0.427-0.638)	<0.001
PNI								
≥45	1		1		1		1	
<45	1.648 (1.317-2.062)	<0.001	1.282 (1.020-1.610)	0.033	1.680 (1.321-2.136)	<0.001	1.343 (1.051-1.715)	0.018

Abbreviations, HR: hazard ratio; CI: confidence interval.

**Figure 3:**
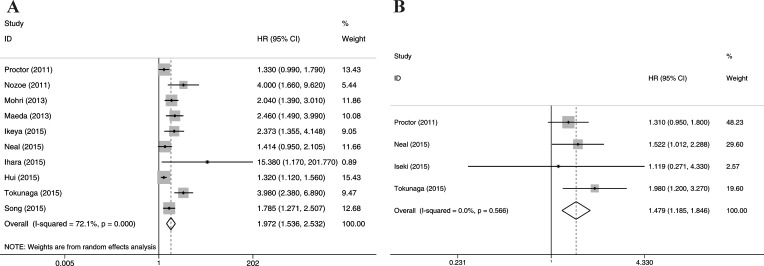
Meta-analysis of the association between low prognostic nutritional index and survival in CRC **A.** overall survival; **B.** cancer-specific survival.

In the patients from the meta-analysis, the pooled result indicates that low PNI was significantly associated with poor OS (HR = 1.972, 95% CI = 1.536-2.532, *P* < 0.001, Figure 3A) and CSS (HR = 1.479, 95% CI = 1.185-1.846, *P* = 0.001, Figure 3B). Further subgroup analysis indicates that the prognostic value of PNI for OS was not undermined by subgroup analysis on the basis of geographical region, surgery, TNM stage, cut-off value, sample size, or study quality (Table S2).

### Optimal cut-off values

**Figure 4:**
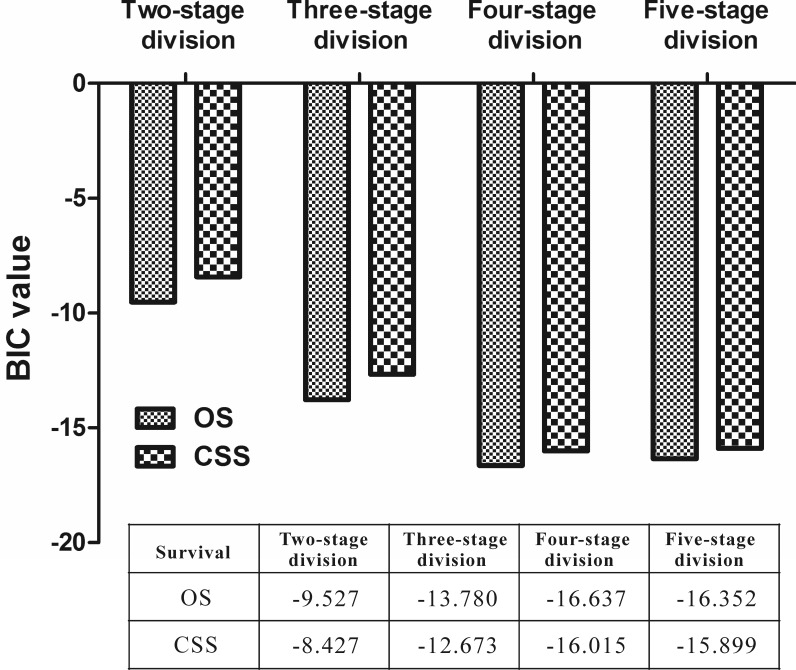
The smallest Bayesian Information Criterion values of overall survival and cancer-specific survival for a different number of stage divisions (from two to five divisions)

**Figure 5:**
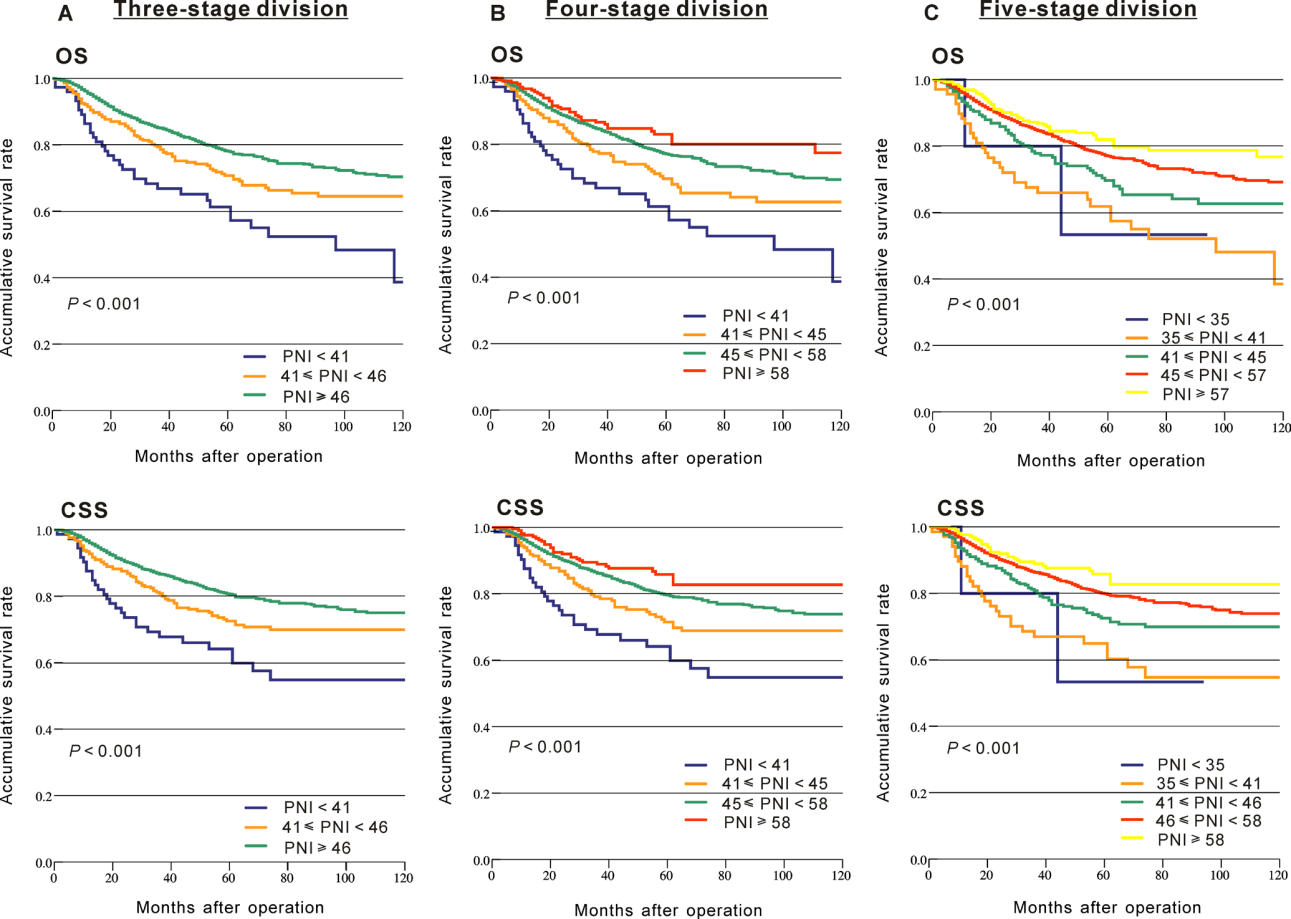
Kaplan-Meier analysis of the overall survival (OS) and cancer-specific survival (CSS) for a different number of stage divisions which indicated smallest Bayesian Information Criterion value (two-stage division see Figure 1) **A.** 41-46 three-stage division; **B.** 41-45-58 four-stage division; **C.** five-stage division (OS, 35-41-45-57 four-stage division; CSS, 35-41-46-58 four-stage division)

To date, the optimal PNI cut-off values and the division of groups by PNI staging remain unknown. In this study, we calculated BIC values for different cut-off values and a different number of stage divisions (from two to five divisions). Our results indicate that BIC values from the 41-45-58 four-stage division (PNI < 41, 41 ≤ PNI < 45, 45 ≤ PNI < 58, and PNI ≥ 58) were the smallest for OS and CSS (Figure 4, Table S3). The Kaplan-Meier curves for different stage divisions, which indicate the smallest BIC values, are shown in Figure 5.

### Evaluation of the prognostic capacity of TNM staging combined with PNI

We calculated the BIC values of TNM staging combined with PNI and of TNM staging alone. The BIC value of TNM staging combined with the 41-45-58 four-stage division was smaller than that of TNM staging alone (−325.76 *vs*. −310.80).

## DISCUSSION

We retrospectively analyzed a large sample of patients from the CMU-SO. Low PNI was an independent prognostic factor for poor OS and CSS and was significantly associated with advanced tumor features such as older age, larger tumor size, and advanced TNM stages. In addition, we retrospectively evaluated the patients from the eleven studies included in the meta-analysis. To the best of our knowledge, this meta-analysis is the first study to systematically assess the prognostic value of PNI and the association between PNI and clinicopathological features in patients with CRC. The meta-analysis results were similar to those of the CMU-SO study, demonstrating that low PNI was significantly associated with poor outcomes and advanced tumor features in CRC.

There are several possible explanations for the association between low PNI and poor prognosis in CRC. First, lymphocytes and serum albumin are significantly associated with prognosis of cancer patients [7, 18, 19]. PNI reflects the nutritional and immune condition of patients because it is based on the peripheral lymphocyte count and serum albumin levels. For this reason alone, PNI correlates with the prognosis of cancer patients. Second, our results indicate that low PNI in CRC patients was significantly associated with advanced tumor features and occurrence of postoperative complications. The significant relationship between postoperative complications and poor prognosis of CRC was confirmed by other studies [20, 21]. Therefore, this relationship may partly explain the association between low PNI and poor survival of patients with CRC. Third, poor immune and nutritional status may lead to a delay in postoperative adjuvant therapy or even abandonment of treatment. Indeed, the results of the CMU-SO study and meta-analysis indicate that significantly more patients in the low-PNI group did not receive postoperative chemotherapy compared with those in the high-PNI group. However, whether PNI should influence the clinical decision for postoperative adjuvant therapy remains to be determined.

In the meta-analysis, the result of the subgroup analysis indicates that low PNI was significantly associated with poor OS at all TNM stages. However, for the patients from the CMU-SO study, OS was significantly different between the low-PNI and high-PNI groups at stages II and III but not at stages I and IV. CSS was significantly different between the groups at stages I and II but not at stages III and IV. We observed that low PNI tended to be associated with poor OS at stage I (*P* = 0.101) and with poor CSS at stage III (*P* = 0.058) and that there were only 44 patients at stage IV in the CMU-SO study. Large-scale studies are necessary to confirm this result.

In this meta-analysis, the PNI cut-off value for the included studies varied between 40.0 and 45.5 (median: 45.0). Kanda et al. [12] reported that a PNI value greater than or equal to 50 was regarded as normal, a value smaller than 50 indicated mild malnutrition, a value smaller than 45 indicated moderate to severe malnutrition, and a value smaller than 40 indicated serious malnutrition. On the other hand, a few studies [15, 22] used receiver operating characteristic (ROC) curve analysis, and another [17] used classification and regression tree analysis to identify the optimal cut-off value; Fu et al. [13] used the Cut off Finder software to determine two cut-off values and divide patients into three groups. Therefore, the optimal cut-off value and the division of the groups by PNI staging remain unclear. In this study, BIC, which is one of most common methods for evaluating the predictive capacity of disease staging, was used to determine cut-off values and the number of stages. We calculated the BIC values for the cut-off values and stage divisions (from two to five divisions). The BIC value in the 41-45-58 four-stage division was the smallest and obviously lower than that of the two-stage and three-stage divisions. In addition, the increased number of divisions such as the five-stage division did not make the BIC value lower than that of the four-stage division and may result in increased complexity of staging. Therefore, we concluded that this four-stage division was the most useful system to determine prognosis for the patients from the CMU-SO. However, the cut-off values and stages identified by a single cohort may not apply to other independent cohorts. Therefore, these results need to be confirmed by future studies.

The BIC value for the combination of TNM staging and PNI in the 41-45-58 four-stage division was smaller than that of TNM staging alone, indicating that TNM combined with PNI in the four-stage division was a better division than TNM staging alone. Therefore, PNI complements the currently used TNM staging system and may increase the accuracy of prognosis for patients with CRC.

There were several limitations in our study. First, our dataset was collected retrospectively from a single institution and the studies included in the meta-analysis were retrospective. Second, all patients from the CMU-SO study were Chinese and most patients from the meta-analysis were from Asian countries; whether the results of this study can be applied to other populations remains unknown.

In conclusion, low PNI was a poor prognostic indicator and was significantly associated with clinicopathological features in patients with CRC. The 41-45-58 four-stage division may be a suitable PNI staging classification to determine prognosis of patients with CRC. PNI may serve as a supplementary index based on the current TNM staging system in CRC.

## MATERIALS AND METHODS

### Patients from the CMU-SO

Information on one cohort of patients with CRC who underwent primary tumor resection at the CMU-SO from March 1995 to May 2014 was collected retrospectively. The patients were selected on the basis of the following criteria: (1) CRC was based on pathological examination; (2) patients had not taken neoadjuvant therapy or anti-inflammatory medications before surgery; (3) laboratory data were obtained before surgery, and patients with synchronous or metachronous tumors were excluded. A total of 2,062 patients were included in this study. Follow-up was completed for all patients by September 2015. The median follow-up was 50 months (range of 1-185). Clinicopathological features, including age, sex, tumor size, tumor location, macroscopic type, differentiation grade, TNM stage, and preoperative laboratory data, were obtained from the medical records of the patients. PNI was calculated as 10 × albumin level (g/dl) + 0.005 × total lymphocyte count (per mm^3^) [8, 9]. The CRC stage was classified according to the seventh edition of the AJCC/UICC TNM classification system.

### Literature search and meta-analysis

We used the search terms “prognostic nutritional index” and “colon cancer/rectal cancer/colorectal cancer” to perform a literature search in PubMed, Embase, and Web of Science databases up to December 31, 2015. Eligible studies were selected on the basis of the following criteria: (1) diagnosis of CRC was based on histopathologic examination; (2) clinicopathological or/and prognostic values of preoperative PNI in CRC were reported; (3) outcome measures were extracted directly or estimated from the studies indirectly; (4) PNI was calculated as 10 × albumin level (g/dl) + 0.005 × total lymphocyte count (per mm^3^). Finally, we included 11 cohort studies [16, 17, 23–31] comprising 3,788 patients in the meta-analysis (the flow diagram of the study selection procedure was shown in Figure S1). The median sample size was 219 patients (range of 80-1321). Seven studies were from Japan, two from the UK, one from China, and one from South Korea. The quality of these studies was assessed using the Newcastle-Ottawa quality assessment scale (NOS) [32]. NOS scores ≥ 6 (median scores of the studies) were assigned as high-quality studies. The characteristics of the included studies are shown in Table S4.

### Statistical analysis

Categorical variables are presented as absolute values and percentages and were compared *via* the chi-square test. Continuous data are expressed as the mean ± standard deviation (SD) and compared using the Mann-Whitney U test. Survival rates, including overall survival (OS) and cancer-specific survival (CSS), were analyzed using the Kaplan-Meier method and compared using the log-rank test. Multivariate analysis was performed using Cox's proportional hazards model. We assessed the predictive capacity of different stage divisions by measuring discrimination, which is the ability to distinguish between high-risk and low-risk patients; we quantified discrimination and determined the cut-off values for the PNI divisions using Bayesian information criterion (BIC) [33]. A smaller BIC value indicates a more desirable stage division for predicting the outcome.

We used hazard ratios (HRs) and 95% confidence intervals (CIs) to evaluate the association between PNI and CRC prognosis. To assess the relationship between PNI and clinicopathological features, odds ratios (ORs) and 95% CIs were used as effect measures. We used the method of Tierney [34] to estimate the HR and 95% CI in the studies in which HR was not reported directly. Cochran's Q test and I^2^ statistics were used to evaluate heterogeneity. I^2^ > 50% or/and *P* < 0.10 indicated a statistically significant heterogeneity, which would allow the use of a random-effect model. Otherwise, a fixed-effect model was used.

Statistical analysis was performed using SPSS software version 20.0 (SPSS, Chicago, IL, USA) and STATA software version 12.0 (Stata Corporation, College Station, TX, USA). *P*-values < 0.05 were considered statistically significant.

## SUPPLEMENTARY MATERIAL


