# Supplementary material for: Prognostic significance of preoperative prognostic nutritional index in colorectal cancer: results from a retrospective cohort study and a meta-analysis
Source: Oncotarget. 2016 Jun 17;7(36):58543–52. doi: 10.18632/oncotarget.10148 (PMC5295450; doi:10.18632/oncotarget.10148)
Supplement: Supplementary file 1 [file oncotarget-07-58543-s001.pdf]

# Prognostic significance of preoperative prognostic nutritional index in colorectal cancer: results from a retrospective cohort study and a meta-analysis

**Supplementary Material**

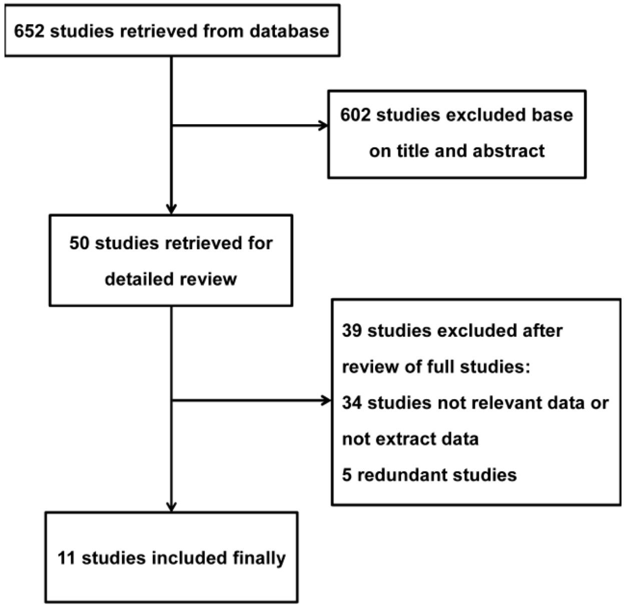


**Figure S1** Flow diagram of study selection procedure for meta-analysis

Table S1 Result of meta-analysis for relationship between low PNI and clinicopathologic features in colorectal cancer

| Subgroup analysis | N | Patients | Pooled OR (95%CI) | *P* | Heterogeneity | | Model used |
|---|---|---|---|---|---|---|---|
| | | | | | $I^2$ (%) | Ph | |
| Age | 3 | 988 | 4.93 (3.454, 6.699)***** | <0.001 | 44.8 | 0.163 | FEM |
| Tumor location (Colon vs. Rectum) | 6 | 2480 | 1.109 (0.932, 1.320) | 0.243 | 35.3 | 0.172 | FEM |
| Depth of tumor (pT3/4 vs. pT1/2) | 2 | 1540 | 3.333 (0.592, 18.76) | 0.172 | 68.3 | 0.076 | REM |
| Lymph node metastasis (Positive vs. Negative) | 4 | 2300 | 1.339 (1.114, 1.161) | 0.002 | 0 | 0.898 | FEM |
| TNM stage (III/IV vs. I/II) | 4 | 2461 | 1.498 (1.268, 1.769) | <0.001 | 27.3 | 0.248 | FEM |
| Histology (Well/moderate vs. Poor) | 5 | 2541 | 1.515 (0.884, 2.597) | 0.131 | 64.6 | 0.023 | REM |
| Postoperative complications (Positive vs. Negative) | 4 | 1225 | 1.369 (1.062, 1.766) | 0.015 | 0 | 0.558 | FEM |
| Postoperative chemotherapy (Yes vs. No) | 2 | 304 | 0.373 (0.187, 0.744) | 0.005 | 32 | 0.225 | FEM |

Abbreviations, 95%CI: 95% confidence interval; FEM: Fixed-effect model; N: number of studies; OR: odd ratio; Ph: *P* value of Q test for heterogeneity test; REM: Random-effect model.

***** Weighted mean difference.

Table S2 Results of overall and subgroup analyses in meta-analysis for effects of PNI on overall survival in colorectal cancer

| Categories | N | Patients | Pooled HR (95%CI) | *P* | Heterogeneity | | Model used |
|---|---|---|---|---|---|---|---|
| | | | | | $I^2$ (%) | Ph | |
| Overall survival (OS) | 10 | 3584 | 1.972 (1.536, 2.532) | <0.001 | 72.1 | <0.001 | REM |
| Region | | | | | | | |
| Asian countries | 8 | 2908 | 2.289 (1.643, 3.187) | <0.001 | 76.4 | <0.001 | REM |
| Non-Asian countries | 2 | 676 | 1.359 (1.072, 1.724) | 0.011 | 0 | 0.809 | FEM |
| Treatment | | | | | | | |
| Surgery | 7 | 2953 | 2.220 (1.517, 3.247) | <0.001 | 78.9 | <0.001 | REM |
| non-surgery | 2 | 257 | 1.927 (1.442, 2.576) | <0.001 | 0 | 0.394 | FEM |
| TNM Stage | | | | | | | |
| I | 2 | 376 | 1.443 (1.088, 1.914) | 0.011 | 0 | 0.787 | FEM |
| II | 2 | 640 | 1.594 (1.259, 2.017) | 0.002 | 36 | 0.211 | FEM |
| III | 2 | 518 | 1.616 (1.226, 2.130) | 0.001 | 8.5 | 0.296 | FEM |
| IV | 7 | 1092 | 1.853 (1.356, 2.533) | <0.001 | 50.6 | 0.059 | REM |
| Cut-off value | | | | | | | |
| ≥45 | 6 | 3095 | 1.718 (1.324, 2.228) | <0.001 | 74.2 | 0.002 | REM |
| <45 | 4 | 489 | 2.692 (1.921, 3.774) | <0.001 | 0 | 0.413 | FEM |
| Sample size | | | | | | | |
| ≥200 | 6 | 3137 | 1.861 (1.351, 2.564) | <0.001 | 78.4 | <0.001 | REM |
| <200 | 4 | 447 | 2.091 (1.631, 2.683) | <0.001 | 20.1 | 0.289 | FEM |
| Study quality | | | | | | | |
| ≥6 | 6 | 2863 | 2.125 (1.465, 3.083) | <0.001 | 84.4 | <0.001 | REM |
| <6 | 4 | 721 | 1.777 (1.230, 2.568) | 0.002 | 55.5 | 0.081 | REM |

Abbreviations, 95%CI: 95% confidence interval; FEM: Fixed-effect model; HR: hazard ratio; N: number of studies;
Ph: *P* value of Q test for heterogeneity test; REM: Random-effect model.

Table S3 the five smallest BIC values for different cut-off values and different number of stages divided

| **Survival** | **Two-stage division** | | | **Three-stage division** | | | **Four-stage division** | | | **Five-stage division** | | |
|---|---|---|---|---|---|---|---|---|---|---|---|---|
| | Cut-off | BV | N | Cut-off | BV | N | Cut-off | BV | N | Cut-off | BV | N |
| **OS** | 45 | -9.527 | 275/1787 | 41-46 | -13.780 | 74/296/1692 | 41-45-58 | -16.637 | 74/201/1571/216 | 35-41-45-57 | -16.352 | 5/69/201/1490/297 |
| | 46 | -8.434 | 370/1692 | 41-45 | -13.774 | 74/201/1787 | 41-45-57 | -16.618 | 74/201/1490/297 | 35-41-45-58 | -16.316 | 5/69/201/1571/216 |
| | 41 | -7.461 | 74/1988 | 41-47 | -12.171 | 74/381/1607 | 41-45-59 | -16.537 | 74/201/1641/146 | 34-41-45-57 | -16.270 | 3/71/201/1490/297 |
| | 47 | -6.152 | 455/1607 | 45-59 | -12.096 | 275/1641/146 | 41-46-58 | -16.285 | 74/296/1476/216 | 34-41-45-58 | -16.245 | 3/71/201/1571/216 |
| | 44 | -5.288 | 213/1849 | 45-58 | -12.043 | 275/1571/216 | 41-46-59 | -16.261 | 74/296/1546/146 | 36-41-45-57 | -16.183 | 6/68/201/1490/297 |
| **CSS** | 45 | -8.427 | 275/1787 | 41-46 | -12.673 | 74/296/1692 | 41-45-58 | -16.015 | 74/201/1571/216 | 35-41-46-58 | -15.899 | 5/69/296/1476/216 |
| | 46 | -7.822 | 370/1692 | 41-45 | -12.297 | 74/201/1787 | 41-46-58 | -15.982 | 74/296/1476/216 | 35-41-45-58 | -15.849 | 5/69/201/1571/216 |
| | 41 | -6.305 | 74/1988 | 45-59 | -12.020 | 275/1641/146 | 41-46-59 | -15.936 | 74/296/1546/146 | 35-41-46-59 | -15.808 | 5/69/296/1546/146 |
| | 47 | -4.465 | 455/1607 | 45-58 | -11.979 | 275/1571/216 | 41-45-57 | -15.881 | 74/201/1490/297 | 35-41-45-57 | -15.766 | 5/69/201/1490/297 |
| | 44 | -2.860 | 213/1849 | 45-57 | -11.709 | 275/1490/297 | 41-45-59 | -15.878 | 74/201/1641/146 | 36-41-46-58 | -15.734 | 6/68/296/1476/216 |

Abbreviations, BV: Bayesian Information Criterion value; CSS: cancer-specific survival; N: number of patients for very stage; OS: overall survival.

Table S4 Baseline characteristics of included studies in the meta-analysis

| Author | Year | Country | Number (Male/Female) | Mean/median Age (Range) | Cut-off | Follow-up median (Range) | Surgery | Staging of (TNM or Duke) | TNM edition | Outcome | SQ* |
|---|---|---|---|---|---|---|---|---|---|---|---|
| Proctor[31] | 2011 | UK | 374 (NR) | NR | 45 | NR | NR | DUKE A-D | NR | OS,CSS | 5 |
| Nozoe[30] | 2011 | Japan | 219 (126/93) | H:69.8±11.6 L:74.5±8.8 | 40 | 2-86 | SR | I/II/III/IV | 7th UICC | OS | 7 |
| Mohri[16] | 2013 | Japan | 365 (223/142) | NR | 45 | 16-83.2 | SR | I/II/III/IV | 7th UICC | OS | 7 |
| Maeda[29] | 2013 | Japan | 100 (54/46) | 60.4±10.6 | 40 | 24.8 | PR | IV | 7th UICC | OS | 6 |
| Ikeya[27] | 2015 | Japan | 80 (44/36) | 63 (36-80) | 44.5 | NR | N | IV | 7th UICC | OS,PFS | 5 |
| Neal[24] | 2015 | UK | 302 (192/110) | 64.8 (26-85) | 45 | 29.7 (4-96) | M | IV | NR | OS,CCS | 6 |
| Ihara[28] | 2015 | Japan | 90 (56/34) | 64.5 | 40 | NR | PR/CR | IV | NR | OS | 5 |
| Iseki[26] | 2015 | Japan | 204 (112/92) | H:66.37±9.99 L:74.33±8.15 | 40 | NR | CR | II/III | 7th UICC | RFS,CSS | 7 |
| Hui[25] | 2015 | China | 1321 (777/554) | 57.5 (18-91) | 45 | NR | SR | I/II/III/IV | 7th AJCC | OS | 7 |
| Tokunaga[17] | 2015 | Japan | 556 (330/226) | H:65.6±11.9 L:69.7±12.7 | 45.5 | 31.8 (1-104） | SR | I/II/III/IV | 7th UICC | OS, CSS | 7 |
| Song[23] | 2015 | South Korea | 177 (83/94) | 52 (25-81) | 45.3 | NR | N | IV | NR | OS | 5 |

Abbreviations, AJCC: American Joint Committee on Cancer; CR: curative surgery; CSS: cause-specific survival; H: high PNI group; L: low PNI group; M: metastasectomy; N: none of patients received surgery; NR: not report; OS: overall survival; PFS: progression-free survival; PR: palliative resection; RFS: relapse-free survival; SD: standard deviation; SQ: score of study quality; SR: surgical resection; UICC: Union for International Cancer Control.

*Study quality was judged based on the Newcastle-Ottawa Scale.
